# Lead-Free LiNbO_3_ Thick Film MEMS Kinetic Cantilever Beam Sensor/Energy Harvester

**DOI:** 10.3390/s22020559

**Published:** 2022-01-12

**Authors:** Gabriel Barrientos, Giacomo Clementi, Carlo Trigona, Merieme Ouhabaz, Ludovic Gauthier-Manuel, Djaffar Belharet, Samuel Margueron, Ausrine Bartasyte, Graziella Malandrino, Salvatore Baglio

**Affiliations:** 1 Dipartimento di Scienze Chimiche, Università Degli Studi di Catania, v.le A. Doria 6, 95125 Catania, Italy; graziella.malandrino@unict.it; 2 INSTM UdR Catania, v.le A. Doria 6, 95125 Catania, Italy; 3 Institut FEMTO-ST, Université Bourgogne Franche-Comté CNRS UMR 6174, 25000 Besançon, France; giacomo.clementi@femto-st.fr (G.C.); merieme.ouhabaz@femto-st.fr (M.O.); ludovic.gauthier@femto-st.fr (L.G.-M.); djaffar.belharet@femto-st.fr (D.B.); samuel.margueron@femto-st.fr (S.M.); ausrine.bartasyte@univ-fcomte.fr (A.B.); 4 Dipartimento di Ingegneria Elettrica Elettronica e Informatica, Università degli Studi di Catania, v.le A. Doria 6, 95125 Catania, Italy; carlo.trigona@unict.it (C.T.); salvatore.baglio@unict.it (S.B.)

**Keywords:** lead-free transducers, LiNbO_3_, MEMS process, energy converters

## Abstract

In this paper, we present integrated lead-free energy converters based on a suitable MEMS fabrication process with an embedded layer of LiNbO_3_. The fabrication technology has been developed to realize micromachined self-generating transducers to convert kinetic energy into electrical energy. The process proposed presents several interesting features with the possibility of realizing smaller scale devices, integrated systems, miniaturized mechanical and electromechanical sensors, and transducers with an active layer used as the main conversion element. When the system is fabricated in the typical cantilever configuration, it can produce a peak-to-peak open-circuit output voltage of 0.208 V, due to flexural deformation, and a power density of 1.9 nW·mm^−3^·g^−2^ at resonance, with values of acceleration and frequency of 2.4 g and 4096 Hz, respectively. The electromechanical transduction capability is exploited for sensing and power generation/energy harvesting applications. Theoretical considerations, simulations, numerical analyses, and experiments are presented to show the proposed LiNbO_3_-based MEMS fabrication process suitability. This paper presents substantial contributions to the state-of-the-art, proposing an integral solution regarding the design, modelling, simulation, realization, and characterization of a novel transducer.

## 1. Introduction

With the advent of smart systems, integrated measurement architectures, and disseminated sensing devices, novel solutions and transduction elements have emerged as an area of incredible impact, potential, and growth [1,2,3]. These solutions have arisen interest, especially considering the following prerogatives: high performance, lead-free, the possibility of incorporating self-generating materials, low cost, miniaturized [4].

New materials, intelligent sensors, and advanced conversion principles are also highly interesting in the context of sensors, actuators, and converters, i.e., technologies such as systems able to convert the energy source nature. For example, the ability to transduce environmental/external energy sources into electrical power, energy harvesting, and certain measurements basics [5,6,7].

This latter family of devices finds recent applications in various areas, including automation, robotics, automotive, biomedical, structural monitoring, and cultural heritage [8,9]. The common feature of such devices concerns the strategies to convert the input energy into corresponding output electrical energy. Literature reports several energy harvesters/converters capable of operating in the presence of various sources of energy, including solar, wind, thermal, RF, and vibrational [10]. The energy storage on a capacitor is typically the common aspect of all the above approaches.

Kinetic energy represents an interesting source, with an abundant and significant amount available in the environment. Examples are induced movements, vibrating equipment, environmental kinetic vibrations, noisily and periodic sources [11,12]. Several transduction principles can be considered to convert these kinetic energy sources into electrical energy (i.e., electrostatic, electromagnetic, magnetoelectric, etc.). It is worth noting that various dynamics [12] have been implemented to improve the performance in macro and micro scale devices and Microelectromechanical Systems (MEMS) [13] by using periodic, noisily, or a mix of waveforms as kinetic energy sources [12,13,14].

Regarding energy converters based on self-generating materials, it should be noted that literature presents various intriguing solutions, such as lead zirconate titanate (Pb(Zr,Ti)O_3_, PZT), aluminum nitride (AlN), polyvinylidene difluoride (PVDF), and, more generally, Electro Active Polymers (EAPs) and compounds.

EAPs based on ions (IEAPs), such as ionic polymer–metal composites (IPMCs), are also widely used as energy converters. This class of compounds can produce electrical power when suitably deformed, and the transduction capabilities rely on ion migration [15]. These devices present various configurations, sizes, and geometries. IEAPs have raised great interest from the scientific community since they are flexible and capable of large deflections, producing few volts as open circuit output. However, they do not lend themselves to integrated solutions and present low power generation capability. In ref. [16], the authors propose IPMCs as an energy converter for harvesting applications. The power generation expected values are in the order of nW/m/s^2^, with samples in the order of centimeters. It is worth mentioning that these macroscale devices’ features are correlated with the specific geometry, input level, transduction elements, and material composition. Nevertheless, it is fascinating given various applications (i.e., automotive biomedical, structural monitoring, disseminated measurement systems), the conception of smaller-scale devices, including integrated systems, miniaturized mechanical and electromechanical elements, microscale approaches, and MEMS-based kinetic energy transducers.

In this context, to decrease the dimension, integrated scale devices, MEMS, and micromachined transducers must be accounted for using other types of materials. Several integrated solutions of beams, bridges, oscillators, and vibrating systems that convert kinetic energy from the environment into electrical power can also be found in the literature.

Jeon et al. [17] have presented a MEMS power generator with a transverse mode based on PZT thin film to convert kinetic into electrical energy. A spin-coated thin film of PZT is used as a conversion layer. A cantilever with a proof mass configuration has been designed as a resonant transducer. An interdigitated Pt/Ti top electrode has been realized to contact the active material with a d_33_ mode to improve the proposed device performance. A 14 kHz resonant frequency has been obtained, and with the presence of a tip, displacements of approximately 5 µm. An output power of around 1 µW, and a DC voltage of around 2.4 V, have been detected. The optimal load corresponds to 5.2 MΩ.

In ref. [18], the authors have proposed another MEMS-based piezoelectric converter solution with an aerosol deposited active layer thin film. The mechanical resonance corresponds to 256 Hz, and in the presence of a tip, displacements of approximately 10 µm are observed. An output power of around 2 µW has been generated. The corresponding output voltage, evaluated with an optimal resistive load of 150 kΩ, is 1.6 V_p-p_. It should be noted that the previously proposed solutions involve post-processing procedures and dedicated steps of a process to include active layers. More recently, PZT epitaxial thin films have been deposited on silicon and structured as cantilevers with tip mass [19]. These MEMS devices could attain 0.27 V/g for an optimal load of 5.6 kΩ and a resonance frequency of 2.3 kHz.

Muralt et al. [20] have reported a MEMS power generator used as a vibration energy harvester with a 2 µm thick PZT on 5 µm of silicon. In the presence of vibrations of about 0.3 µm, at 870 Hz, maximum power of about 500 nW is generated. The output voltage corresponds to about 0.9 V. The optimal resistive load is about 10 kΩ.

Based on their high-performances, current devices tend to be developed using lead-based PZT technologies and derivatives. Due to their classification as harmful materials, related to their toxicity, lead-free replacements are needed. These proposed devices comply with the directives based on the Restriction of Hazardous Substances Directive (RoHS) thanks to lead-free materials.

Various papers have been proposed in the literature regarding integrated solutions with embedded lead-free active layers used as conversion elements. Technologies currently in use, including commercial ones, are developed with processes-based zinc oxide (ZnO) and AlN layers. Regarding ZnO, it has been implemented in piezoelectric MEMS vibration energy harvesters with two piezoelectric elements for higher output performance, where the energy harvester has been fabricated on a Si wafer by means of standard micro-machining techniques [21]. The resonance frequency is 1.31 kHz, achieving 1.25 μW for 1 g acceleration level.

In ref. [22], the authors exploit the performance of an AlN-based MEMS as a kinetic to the electric converter. A commercial MUMPS process, based on 0.5 µm AlN, has been used. In particular, the performance as a sensing device has also been pursued, showing the generation of about 2.5 mV voltage with an acceleration level of 0.6 m/s^2^. In the perspective of being used as a harvester, this embedded material performance is less than some nW. In ref. [23], AlN microstructure cantilevers were implemented in an integrated energy harvesting module with a power management circuit, obtaining 1.4 nW with a resonance frequency of 1.5 kHz.

LiNbO_3_ shows the highest material coupling factor among lead-free piezoelectric materials such as AlN, PVDF, and ZnO and, because of its Curie temperature (1100 °C), it opens the possibility to high-temperature applications [24,25]. For these reasons, the pursued approach has arisen interest in order to fabricate microscale sensors and transducers. Moreover, macroscale LiNbO_3_ energy harvesters have been recently investigated, obtaining power densities comparable with lead-free and lead-based materials [26]. Therefore, their use has been proposed to power up macroscale devices [27]. On the other hand, their role in powering the Internet of Things (IoT) nodes has also been explored, either by implementing them with off-the-shelf radio frequency modules, showing the possibility of sending data wirelessly every 2 s at resonance [28], or using their electromechanical properties along with low-power Bluetooth modules to obtain self-powered acceleration sensors [29].

This paper improves the state-of-the-art; in particular, the novelty concerns the design, modelling, simulation, realization, and characterization of an integrated lead-free energy converter based on a suitable MEMS fabrication process with an embedded layer of LiNbO_3_. To the best of the authors’ knowledge, no technology is yet available, nor MEMS fabrication process has been reported, on lead-free integrated kinetic energy converters based on LiNbO_3_. Our lead-free fabrication technology has been developed to obtain micromachined resonators able to convert kinetic energy into electrical energy. The process proposed here presents several features with the possibility to fabricate MEMS scale devices, integrated systems with miniaturized mechanical and electromechanical sensors, and transducers with lead-free active layer used as the main element of conversion. When the system is fabricated in the typical cantilever configuration, it produces an output voltage signal due to flexural deformation.

The proposed lead-free LiNbO_3_ converter can generate a voltage with amplitude higher than diode thresholds (>200 mV), where the electromechanical transduction capability is exploited for sensing and power generation/harvesting applications. Theoretical considerations, simulations, numerical analyses, and experiments are presented to show the proposed LiNbO_3_-based fabrication process suitability.

## 2. Theoretical Considerations

**Figure 1:**
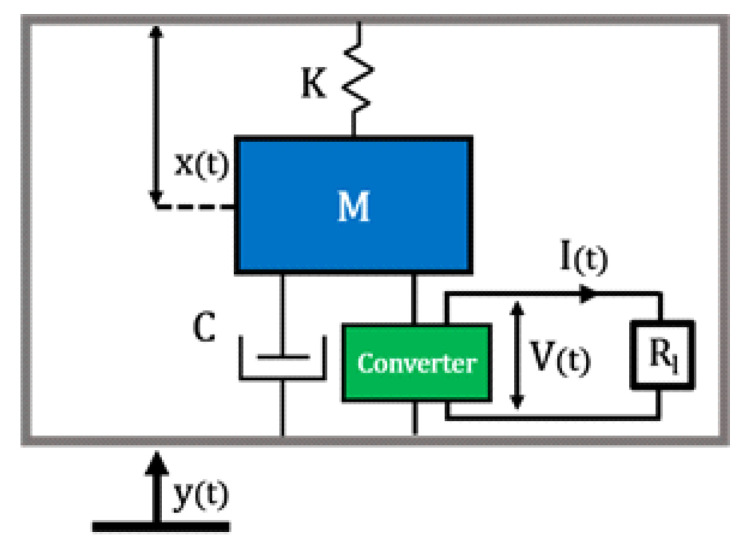
Schematics of the kinetic energy converter.

The equivalent circuit of the device is a system that considers the behavior of the LiNbO_3_ element and the natural mechanical frequency as a resonator with a single degree of freedom [30]. In this approximation, the converter can be modeled as an electromechanical system composed of an inertial mass M, a damper C (mechanical losses), and a spring K (stiffness of the mechanical structure), that undergoes the action of an external driving force $M\ddot{y}$, see Figure 1.

**Figure 2:**
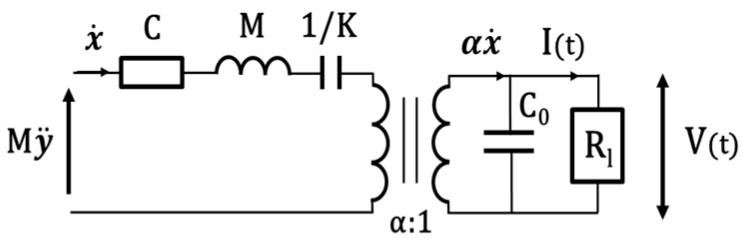
Equivalent electrical circuit.

The relative displacement of the inertial mass concerning the reference frame is then x, and the current I is proportional to the velocity $\dot{x}$, electromechanically converted by a transformer with a ratio α:1, where α is the electromechanical force factor, see Figure 2.

Therefore, the LiNbO_3_ element is a current generator connected in parallel to the clamped capacitance C_0_, where V represents the voltage measured on the resistive load R_l_. It is possible to describe such a system in terms of the following coupled equations:

(1) $$\{\begin{array}{c} M\ddot{x}=M\ddot{y}-Kx-\alpha V-C\dot{x}\\ I=\alpha \dot{x}-C_{0}\dot{V} \end{array}$$

with this formalism, we can express the electromechanical coupling factor k^2^ and the damping ratio from mechanical losses ζ as:

(2) $$k^{2}=\frac{\alpha^{2}}{\alpha^{2}+KC_{0}}$$

(3) $$\zeta =\frac{C}{2\sqrt{KM}}$$

Therefore, in this electromechanical system, the mechanical losses are represented by ζ, and the electromechanical coupling factor k^2^ represents the ratio between the input mechanical energy and the output electrical energy, which is always <1. From the mathematical expression of the coupled system, it is possible to evaluate the voltage and power on a resistive load connected in parallel to the converter so that the power would be given by $P=\frac{V^{2}}{{2R}_{l}}$, hence in the explicit form as:

(4) $$P=\frac{1}{2}\frac{R_{l}\omega^{2}\alpha^{2}}{{1+(\omega R}_{l}C_{0}{)}^{2}}{x_{M}}^{2}$$

where the power is then proportional to the angular frequency ω and the displacement magnitude x_M_, thus, the maximum power is given at the resonance angular frequency ω_0_ and optimal resistive load value R_opt_ is expressed as:

(5) $$P_{\max}=\frac{1}{4}\frac{\alpha^{2}}{C_{0}}\omega_{0}{x_{M}}^{2}$$

## 3. Materials and Methods

**Figure 3:**
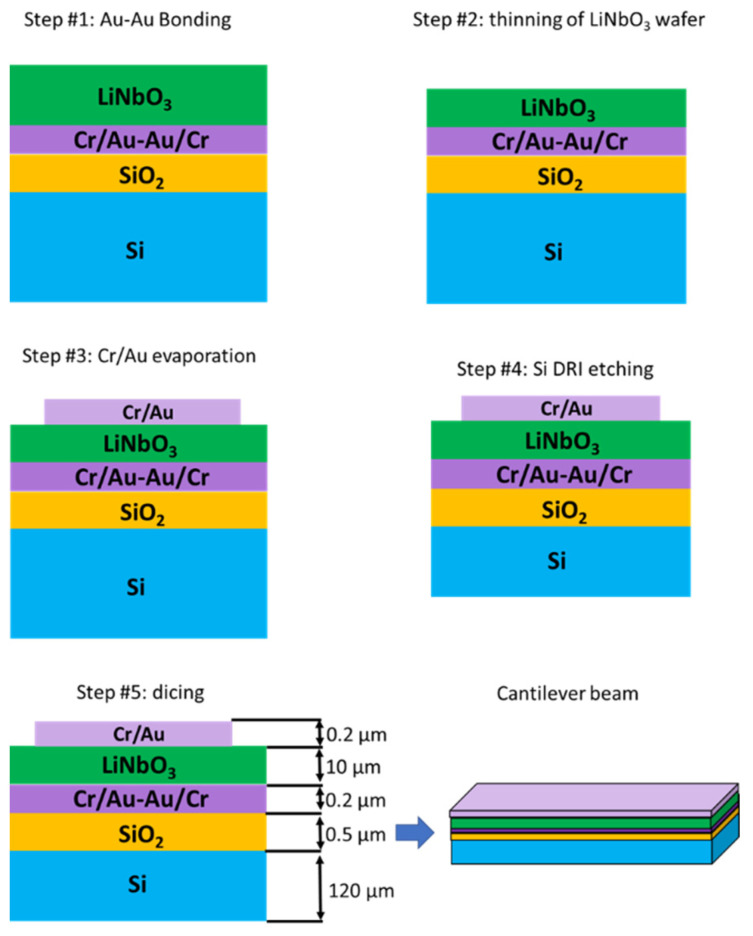
Microfabrication flow chart for cantilever beam development.

As we can see in Figure 3, regarding the microfabrication process, the material stack constituted of lithium niobate (LiNbO_3_), chromium (Cr), gold (Au), silica (SiO_2_), and silicon (Si) is achieved with a standard 500 µm Si/SiO_2_ substrate with an oxide layer of 500 nm and a (YXlt)/163°/90° LiNbO_3_ (following IEEE standard on rotation [31]) single crystal substrate polished in both sides. In both Si/SiO_2_ and polished LiNbO_3_ substrates, an adhesive layer of Cr of 15 nm and a thin layer of Au of 85 nm by sputtering is deposited to perform Au-Au bonding between the substrates. After bonding, the LiNbO_3_ layer is lapped to 12 µm and polished again (TTV 2 µm). After a step of micro-polishing the piezoelectric layer, top electrodes are deposited with the same adhesive layer thickness of Cr, and 185 nm of Au, patterned by UV lithography in the shape needed to create beams. Further procedures are related to mechanical cutting with a 100 µm thick saw and a precision of 2 µm.

**Figure 4:**
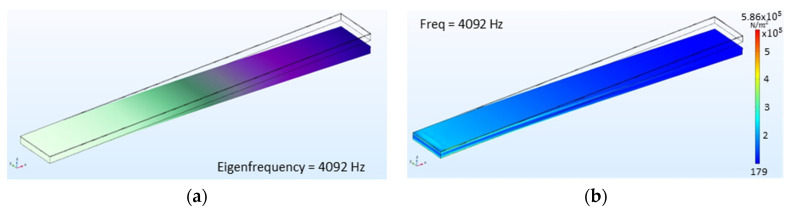
Linear Beam with 6.5 mm length and 800 µm width FEM simulations results: (**a**) first eigenmode with a frequency around 4092 Hz. (**b**) Stress values with a max. value 5.86 × 10^5^ N/m^2^.

Regarding the materials on this matter, the need for a protective sacrificial layer, in this case, a 3 µm resin layer, is needed while cutting with the high precision dicing saw. Because of the devices’ size, processing steps take place to release the devices from the frame. Reactive-Ion Etching (RIE) processes are performed to straighten the mechanical cuts and achieve the beam’s thickness (see cantilever beam in Figure 3). The microfabrication technology has been carefully selected based on standard procedures to foresee the possibility of upscaling, if convenient, for certain applications. Various devices have been developed under the previously described microfabrication procedure to validate the suitability of the LiNbO_3_ MEMS process, such as linear beams. Even though more intricated structures can be more suitable for these applications, linear devices with lengths from 13 mm to 4.5 mm, 800 µm width, and 120 µm thick, have been selected to have as benchmark previous work experience in designing and characterizing MEMS devices with similar behavior, based on commercially available PiezoMUMPs manufacturing technology [32]. In parallel, FEM simulations have been performed to better understand the mechanical behavior. In particular, the case study regards a cantilever beam having a width of 800 µm and a length of 6.5 mm. Simulations have been performed with the software COMSOL Multiphysics and the specific MEMS module, with a solid body representing its weight as a load, a fixed constrain on the extreme cross-section, and a mesh of 1936 prim elements; only consider the theoretical calculation for an optimal setup without any additional disturbances after the ones we can estimate mathematically. A study in frequency has been carried out to approach the value of the first eigenmode and use it to narrow down the physical measurements, while a study in the time domain has allowed us to understand the devices’ stress values and movement behavior, see Figure 4.

**Figure 5:**
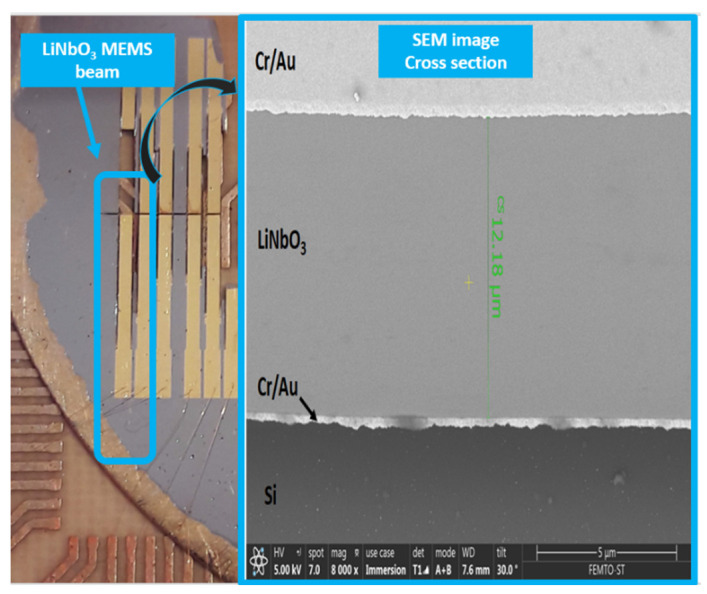
(YXlt)/163°/90° LiNbO_3_-based MEMS energy converters with various lengths (on the **left**) and cross-section of a realized device (on the **right**).

Figure 5 shows the fabricated (YXlt)/163°/90° LiNbO_3_-based MEMS energy converters, fixed in a PCB and wire bonded. A cross-section of a beam is also highlighted.

## 4. Experimental Results and Discussion

**Figure 6:**
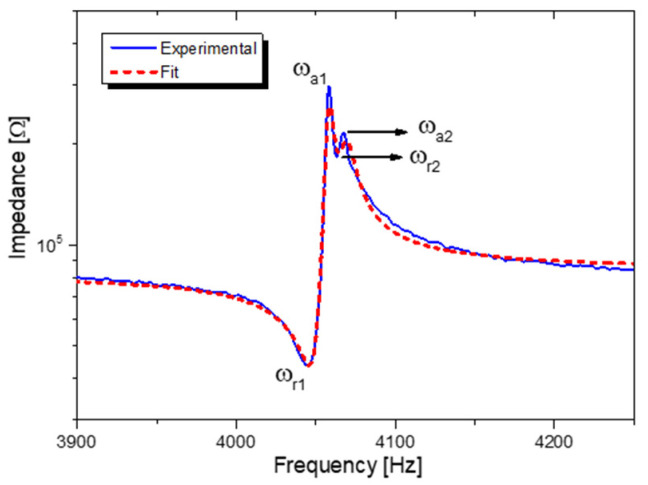
Impedance measurements for the LiNbO_3_ element.

The experimental characterization of the (YXlt)/163°/90° LiNbO_3_ device done with Agilent Technologies E5061B and a test feature Keysight Technologies 16047E, M, C, and K values, have been estimated from resonance and anti-resonance frequency of electrical impedance. The results have shown two different modes, giving two sets of ω_r_ and ω_a_. The experimental data have been fitted to extrapolate ζ and k^2^ of the prototypes. A representation of the data from the impedance measurement is presented in Figure 6.

The first mode estimated mechanical damping factor is ζ = 0.0017, with low dielectric losses (tanδ < 1%). As expected, (YXlt)/163°/90° LiNbO_3_ high-quality single crystal is responsible for the considerably low values of damping and losses. Concerning the electromechanical coupling factor, the converter shows low coupling, the value measured is ${k_{1}}^{2}$ = 0.7%. In the lumped model, the spurious mode has not been considered due to very low coupling (${k_{1}}^{2}$ = 0.04%). The appearance of the spurious mode could be due to the mechanical effect of resonance with other beams that are part of the device or the sample thickness variation. Eventually, given the devices’ low coupling, we could foresee that the electronic interface would not affect the structure displacement [33].

**Figure 7:**
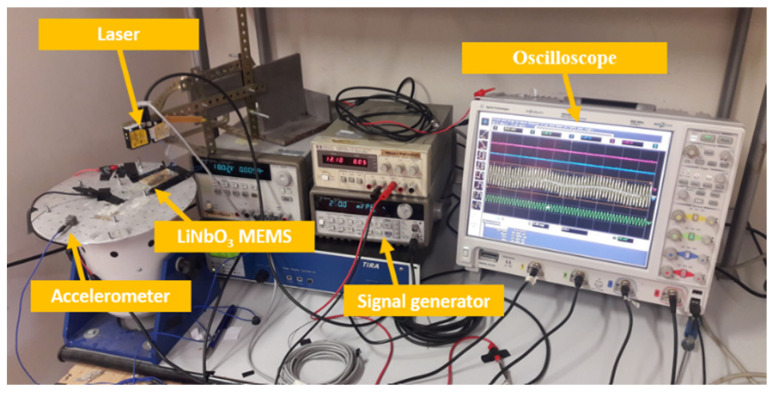
Experimental Setup.

The experimental setup (see Figure 7) used to study the integrated converter is based on:

- A signal generator HP33120A, for impressing a suitable waveform to the shaker;
- An oscilloscope, Agilent MSO9064A, for acquiring the signals;
- A single-axis accelerometer used to monitor the imposed vibrations;
- Two laser sensors, used for measuring the sensor displacement (anchor and tip).

**Figure 8:**
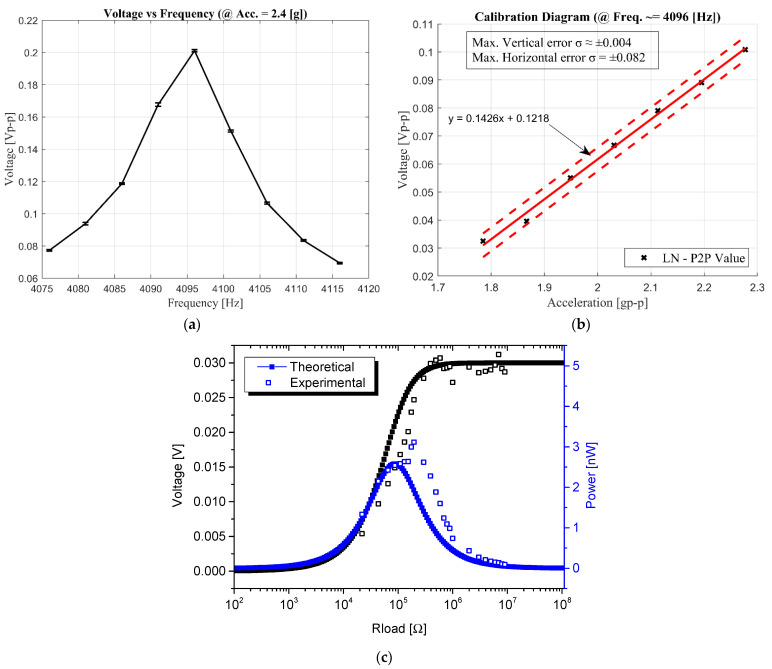
Characterization results of the device: (**a**) voltage vs. frequency (**b**) voltage vs. acceleration–calibration diagram (**c**) Power and voltage output comparison for the simulated and experimental results.

The device was studied considering extensive tests and a metrological characterization. In this context, the frequency response is presented in Figure 8a, where the average of 14 tests each is represented with 9 data points for voltage vs. frequency, showing the resonance frequency, *f*_0_, around 4096 Hz. This value is obtained by sweeping the frequency from 4076 Hz to 4116 Hz with steps of 5 Hz. An open-circuit voltage (V_oc_) of about 0.208 V_p-p_ is found at resonance. Similarly, for voltage vs. acceleration (Figure 8b), the source signal amplitude has been changed to obtain a variation of equidistance steps of 0.082 g of acceleration from 1.785 g to 2.28 g. With a linear fit of seven data points, this figure is represented as a calibration diagram to consider the voltage uncertainty, with a maximum value σ = 0.004 V_p-p_. This result is achieved with a polynomial curve fitting based on Matlab’s polyfit and polyval functions, where polyfit returns the coefficients of a degree *n* polynomial equation ($p\left(x\right)=p_{1}x^{n}+p_{2}x^{n-1}+\cdots p_{n}x+p_{n+1}$), to best fit the curve, and polyval returns the estimated error.

The force factor α has been measured from the displacement and voltage measurements in open circuit conditions. From the V_oc_ measured at resonance and considering a peak-to-peak displacement of 2 µm, the force factor α for the converter has been calculated, while the clamped capacitance C_0_ has been measured at 1 kHz. With the parameters summarized in Table 1, we can approximate the behavior of the cantilever around its resonance frequency; even though being a single degree of freedom model, it does not allow us to consider possible nonlinearities of the system.

Using the formalism presented in Equation (5), we have estimated the power output from the LiNbO_3_ element obtaining about 2.6 nW for a load of approximately 100 kΩ, which results in a discrepancy of 17% between experimental and theoretical data. This difference can be attributed to considering a beam without a tip mass as a single degree of freedom system [34]. The power and voltage simulations from the lumped model, along with the experimental results, are shown in Figure 8c.

Regarding the voltage vs. resistance load, a measurement with resistance in parallel take place for resistance values related to the logarithmic scale, emphasizing the range between 100 kΩ and 1 MΩ where the proper measurements and the performance of the device show a saturation point of 0.03 V_rms_. The power vs. resistance load, with a maximum power over 3 nW_rms_ at an optimal resistance value of 196 kΩ, is derived from the voltage vs. resistance load values, mathematically calculated using Ohm’s Law.

A 96 kΩ discrepancy is observed between experimental and theoretical values. The lumped model approximates the harvester to a single degree of freedom system and does not consider electromechanical interface imperfections such as clamping or thickness variation of the piezoelectric or silicon layer. Moreover, variations in magnitude and slight frequency derivation are present between the measurements.

Nevertheless, an expected behavior can be noticed, promising for future variegate applications. Finally, we have estimated the devices’ power density with an acceleration of 2.4 g, which shows a resulting power output of 1.9 nW·mm^−3^·g^−2^.

Table 2 presents finite element simulations comparing (YXlt)/163°/90° LiNbO_3_ and commonly used ceramic PZT-5A. The geometry and parameters used in the simulation are the same for both materials, and the values are measured in open circuit conditions, assuming a tip displacement of 1.4 µm. Typically, due to the lower stiffness of PZT-5A ceramics, the resonance frequency of the device shifted to a lower value (3536 Hz). However, the voltage output is similar to LiNbO_3_, obtaining a V_rms_ of 33 mV. The power output is similar for LiNbO_3_ and PZT-5A (3 nW and 2 nW, respectively), showing comparable results.

## 5. Conclusions

This paper demonstrates the possibility of using LiNbO_3_ MEMS devices for sensing applications or as energy harvesters. We analyzed the performances of a piezoelectric beam with 6.5 mm in length and 800 µm width, which showed a peak-to-peak open-circuit voltage of 0.208 V with resonance frequency at 4096 Hz. The piezoelectric beam showed low mechanical damping (ζ = 0.0017), a coupling factor k^2^ = 0.7%, and whenever operated at resonance, it attained a power density of 1.9 nW·mm^−3^·g^−2^. Finite element simulations and a single degree of freedom model were used to predict and analyze the experimental results. Further improvements in the sensing capabilities and electromechanical coupling of the devices have been obtained by implementing a silicon tip mass to maximize the stress on the beam and lower its resonance frequency. Moreover, the proposed microfabrication route results are promising, especially for industrial upscaling and for the application of LiNbO_3_ in MEMS scale devices.

## Figures and Tables

**Table 1:** Experimental evaluated lumped model parameters.

C_0_ (pF)	M (mg)	C (N·s·m^−1^)	K (N·m^−1^)	α (N·V^−1^)	X_M_ (µm)
744	1.133	9.948 × 10^−5^	731.4	4.7619 × 10^−5^	0.98

**Table 2:** Simulation of different materials with the same MEMS design.

Material	Frequency (Hz)	V_rms_ (mV)	Power (nW)
(YXlt)/163°/90° LiNbO_3_	4092	37	3
PZT-5A	3536	33	2

## Data Availability

Due to the nature of this research, under the frame of H2020 MSCA-ITN Enhance project grant agreement No. 722496, the data that support the findings are not publicly available. The data of this study are available from the corresponding author, G.B., upon reasonable request.
